# Detecting Undifferentiation of Tertiary and County Hospitals in China in Adoption of DRG Instrument

**DOI:** 10.3390/healthcare9080922

**Published:** 2021-07-21

**Authors:** Yuntian Chu, Hongbing Tao

**Affiliations:** Department of Health Administration, School of Medicine and Health Management, Tongji Medical College, Huazhong University of Science and Technology, Wuhan 430074, China; jimchuy@yahoo.com

**Keywords:** health system, tiered healthcare delivery system, inpatients, referral

## Abstract

(1) Background: Undifferentiated function for medical institutes in different levels had been a barrier to China’s healthcare reform. Thus, this study aimed to detect medical services that were capable offered both in tertiary and county hospitals in China and discuss the process of detection. (2) Method: Data of 2 tertiary hospitals that were city level and 12 county-level hospitals from one city in China were collected and grouped into diagnosis-related groups (DRGs). A strategy with four steps was devised by considering the aspects of service volume, in-hospital mortality rate, in-hospital adverse events rate, and inpatient cost. Additionally, a comparison of each indicator was made between city- versus county-level hospitals. (3) Results: There were no differences in service quality between the two levels of hospitals while county hospitals had lower average inpatient costs in 129 DRGs that covered 39.5% of all cases. About CNY 0.26 billion would be saved if certain cases were paid at county-level prices. (4) Conclusion: The study proposed a strategy with four steps that could help in locating the range of diseases in which patients’ admission suffered from the problem of undifferentiation between hospitals’ functions to reduce the irrational growth of healthcare expenditure.

## 1. Introduction

Regulations limiting the number of patients choosing medical institutions were introduced in many countries and places in order to improve the order of diagnosis and treatment so that patients with less serious conditions seek therapy at general practitioners (GPs) or primary medical institutions and those with more severe conditions visit specialties. One well-known regulation was gatekeeping in the primary care system, which offered general practitioners (GPs) authorizing access to specialty care or hospital care. Patients in countries such as Australia, Canada, Italy, Norway, the United Kingdom, etc. were forced to require GP referral to specialist healthcare services [1]. Patients in Norway who needed acute treatment but not specialty care were even transferred by primary care physicians to municipal acute wards as an alternative to hospitalization [2]. In other countries such as Japan, though patients are not forced to register with a general practitioner or family doctor, they should pay additional costs if bypassing primary care physicians and receiving care in higher-level facilities [3].

The key point is how to decide which patients need a referral to specialty care. In countries applying gatekeepers in the primary care system, referrals to specialty care were commonly determined by GPs, whereas even in these countries, the function and power of gatekeepers were still controversial [1]. For example, some studies declaimed that GPs as gatekeepers would offer lower health services, compared with specialists, and lead to lower expenditure [4]. Other studies regard GP having the power of referral to specialty would delay diagnosis, which exacerbates the waste of medical resources [5].

Chinese citizens are free to receive healthcare services at any institution [6]. Hospitals in urban areas offer their services to the whole city, county, or district, whereas the competitive and for-profit market environment for medical institutions in the past decades formed a fragmented hospital-centric healthcare delivery system and homogenization of medical services offered by different providers. Additionally, the allocation of health resources was irregulated [7]. The public general tertiary hospitals provide almost every kind of service, including general practice for common diseases, long-time hospitalization for treatment of chronic conditions, or rehabilitation. As a result, large-scale general public tertiary hospitals admitted the majority of patients and used the majority of healthcare resources due to their reputation, better environment, and higher quality of care, which usually indicates higher expenditure [6,8].

Policies and regulations were introduced to address the topic of discriminating functions for different kinds of healthcare providers. In 2015, guidelines from the State Council proposed a tiered healthcare delivery system in which health facilities at each level have differentiated functions that restrict the care delivered, in order to replace the hospital-centric approach [9]. Providers would be coordinated or integrated by offering services across levels. The form of medical alliances by institutions in various levels was encouraged to promote the tiered hospital delivery system, many of which were designed to operate as leading hospitals to help improve health service capacity of lower-level hospitals and primary care facilities by training medical staff, sending reputed physicians to outpatient visits at intervals or initiate joint teleconsultations [10,11]. Moreover, “gatekeepers” in the health system were commenced to establish by government acts and plans such as meliorating general physician training system and offering registration of family doctor teams for citizens since 2016 [6].

All of these are policies in place for differentiating functions of healthcare facilities, and providers should refer those patients who did not match their functions. However, little is known about what kind of services these hospitals offered were not different, and how many of the patients made self-referrals incorrectly. The aim of this study was to locate the diseases that nontertiary hospitals could also offer with sufficient quality and lower expenditure and present a strategy of the selection process.

## 2. Materials and Methods

### 2.1. Study Population

Information of inpatients in 2019 from 14 general public hospitals in one city from Shandong Province, China, were collected retrospectively from the City Healthcare Information Platform as the sample of this cross-sectional study. The city’s population is 8.35 million, and it contains 11 county-level administrative districts. There is at least one county-level public hospital in each district, of which two county-level hospitals are located downtown. Additionally, two city-level hospitals are also located downtown. The two city-level hospitals are the only Grade-A tertiary hospitals in the city. Additionally, the referral progress between public general hospitals here generally occurred across the city and county-level hospitals. Few patients were transported between hospitals at the same level.

The front page of Medical Records from inpatients was collected as the sample of this study, including demographic information as age, sex, race, times of inpatient, blood type, newborn age, and weight; codes and names for principal and secondary diagnoses; codes, names, and dates for surgery and operations; other administrative data such as whether visiting by emergency, discharge type and ward, length of stay, and the total amount of in-hospital costs. Fields that may lead to patients’ identity such as ID number or address were not available to our study.

A total of 662,196 cases were collected and were divided into different diagnosis-related groups (DRGs) by Beijing version DRG splitter (BJ-DRG) [12]. After excluding cases with missing or irregulated coding of principal diagnosis, newborn cases without weight information, total in-hospital costs lower than CNY 50 (about USD 7.6), length of stay over 60 days, unmatching codes of diagnosis and operation, diagnosis of pregnancy with age over 50 years old, and palliative treatment malignant tumor cases, 606,623 observations were included and were divided into 750 DRGs.

### 2.2. Selection Strategy

For samples included, four steps of selection were devised for locating cases that were nonsignificantly different in the quality of healthcare services between the two levels of hospitals and had significantly lower costs in the county-level hospitals.

**Figure 1:**
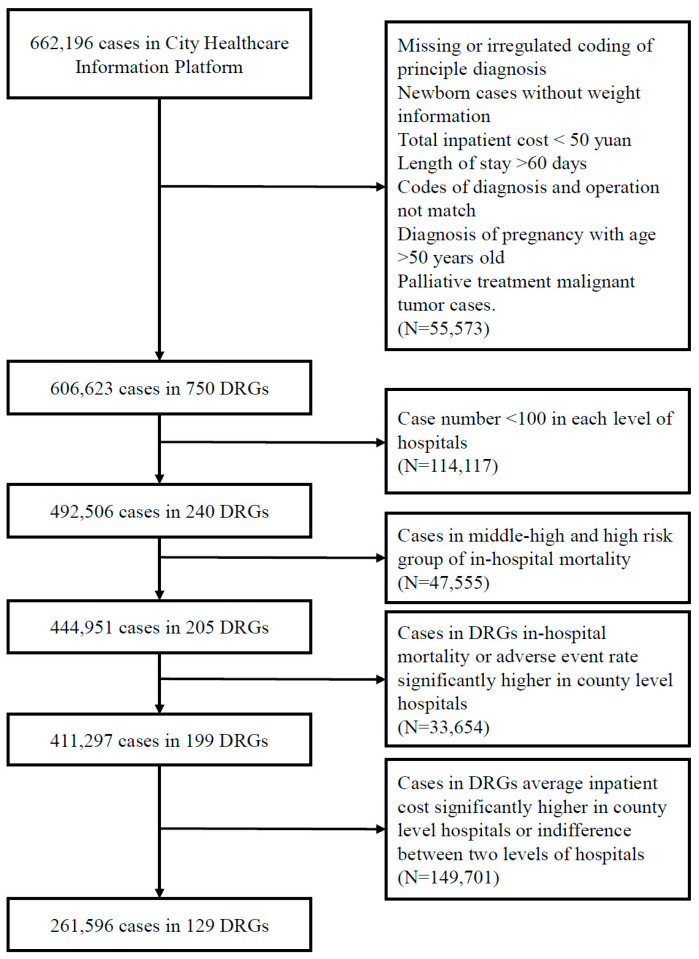
Flowchart for case selection process. DRGs: diagnosis-related groups.

As shown in Figure 1, the first step was to find DRGs with at least 100 cases in one year in both levels, which meant the diseases or conditions in a certain group were not very rare so that comparison between the two levels was essential. The second step was to compare the mortality rate of each risk group between the two levels of hospitals, which implied the difference of medical service quality, and the risk groups with nonsignificant results were selected. The third step was the comparison of service quality within each DRG left, including the rate of in-hospital mortality and adverse events, and cases in DRGs with no difference between county and city hospitals were selected. Additionally, the last step was to locate DRGs in which total inpatient costs were significantly lower in county hospitals in cases where the quality of services was possibly homogenous.

### 2.3. Dividing Diagnosis-Related Groups (DRGs)

All cases of medical record front sheets in this study were divided into DRGs in BJ-DRG version 2014, offered by Shanghai Creating Information Technology Co., Ltd., (Shanghai, China). BJ-DRG covered all kinds of inpatient cases and split cases based on information of diagnosis and operation or procedure codes in the medical record front sheets. Over 20,000 diagnoses from International Classification of Diseases, Tenth Revision, Clinical Modification (ICD-10-CM) and 6000 clinical operations and procedures from International Classification of Diseases, Ninth Revision, Clinical Modification (ICD-9-CM) were matched in BJ-DRG and were grouped based on the similarity of clinic progress and resource consumption.

### 2.4. Outcomes

The in-hospital mortality, adverse events, and total inpatient costs are the primary outcomes of this study. Length of stay was also shown as a baseline characteristic of each institution. The field of discharge type in administrative data of medical records was used to recognize whether a patient died in the hospital. The mortality risk groups were estimated by a logarithm of the in-hospital mortality rate of each DRG, and DRGs were then considered as a zero-mortality group, low mortality risk group, low-middle risk group, high-middle risk group, or high-risk group [12]. Related weight (RW) that considered the level of difficulty and resources consumed for each DRG was evaluated by BJ-DRG at the time when cases were divided into DRGs. Consumption index, which are indicators reflecting the level of cost and length of stay spent for treating similar patients, compared with other institutes, were calculated by standardization of cost and length of stay [12].

Patients having pressure ulcers, complications, or infections caused by operations or procedures, and adverse events resulted from drugs were considered having in-hospital adverse events in our study. Patients with any codes of pressure ulcer in the secondary diagnosis were considered to have pressure ulcers. Patients who were under operation or procedure at this admission with codes of complications after procedure or operation in their secondary diagnosis, excluding those having certain codes in their principle diagnosis, were regarded as occurring complications resulted by operation or procedure. Complications included postoperative hemorrhage or hematoma, metabolic disorder, complications of the respiratory system, urogenital system, nervous system, circulatory system, eyes and appendages, and infection.

Postoperative hemorrhage or hematoma and postoperative infection were defined as cases with certain codes, respectively. Patients with a diagnosis of hypofunction of pituitary, thyroid, or parathyroid after an operation, procedure, or radiotherapy were considered to have a postoperative metabolic disorder. Cases in secondary diagnosis code such as tracheostomy infection, airway obstruction, difficult decannulation after tracheotomy, tracheoesophageal fistular, bronchial anastomotic stenosis, etc. were recognized as respiratory complications. Postoperative complications in the urogenital system contained conditions of uterine incision diverticulum, postoperative urethral fistula, pelvic adhesion, bladder fistula stenosis, vaginal stump bleeding. Complications in the nervous system included cerebral hernia, cauda equina nerve injury, limb dysfunction, intracranial pneumatosis, cerebral vasospasm after angiography. Diagnosis of arteriovenous fistula stenosis, thrombosis, heart failure after heart surgery, cardiac dysfunction after valve replacement, abdominal aortic occlusion, and chylothorax were regarded as complications of the circulatory system. Additionally, patients with vitreous syndrome, shallow anterior chamber, no anterior chamber, or retinal detachment were regarded as having complications of postoperative corneal diseases.

Additionally, the adverse event caused by drugs included drug or chemotherapy-induced myelosuppression, drug-induced thrombocytopenia, hypothyroidism or adrenocortical dysfunction, or drug-induced withdrawal, poisoning, and other mental disorders.

### 2.5. Statistic Analysis

Category variables such as in-hospital death were shown in numbers and percentages. Continuous variables such as cost and length of stay were displayed in median and quantiles. Wilcoxon rank-sum tests were applied for comparison of continuous variables between groups, and chi-square test for categorical variables. *p* values lower than 0.05 were considered to be significantly different for the tests. Statistical analyses were performed using R statistical software, version 3.6.1 (Nokia Networks, Espoo, Finland) (https://www.r-progject.org accessed on 19 July 2021).

## 3. Results

### 3.1. Baseline Characteristics for Hospitals in Sample Area

Table 1 and Table 2 demonstrated the baseline characteristics for the 14 public general hospitals in the sample are in 2019, including the number of inpatients, case mix index (CMI) that reflected the complexity of patients’ conditions in each hospital, in-hospital mortality rate, the average inpatient cost, and length of stay. The city hospitals generally have higher CMI and inpatient costs while having a lower length of stay, whereas the consumption index of cost and stay was at the middle range in the city. No trends of mortality rate were found between hospitals.

### 3.2. Selection of Service Scales of City and County Hospitals

All the cases from the city platform were evaluated by BJ-DRG splitter, and 606,623 observations were divided into 750 DRGs successfully, of which 221,655 cases that covered 746 DRGs were from city-level hospitals, and 384,968 covering 695 DRGs were from county hospitals. For medical records included, DRGs with at least 100 cases this year in both the two levels were chosen and underwent further analysis for the possibility of undifferentiation of services provided from each level of hospitals for these patients. There were 492,506 observations from 240 DRGs selected. Among them, 34.5% were from city hospitals, and 65.5% were from county hospitals. In these cases selected, there were 1553 patients deaths observed, of which 424 occurred at city hospitals with an in-hospital mortality rate of 0.25%, and 1129 occurred at county hospitals with a mortality rate of 0.35%. The differences in mortality rates between the two levels were generally significant (Table 3).

### 3.3. Difference in Mortality Rate in Each Risk Group between the Two Levels

Results of in-hospital mortality rates in different risk groups are also demonstrated in Table 3. Additionally, the difference between the two levels of hospitals was tested excluding the zero-mortality group. In low and low-middle risk groups, the difference between the two levels was not significant, whereas, in high-middle and high-risk groups, mortality rates in county hospitals were significantly higher than in city hospitals, indicating that for patients who were in high-middle and high-risk groups, the quality of services from county hospitals was still far from that of city hospitals.

Despite this result, the average inpatient costs were significantly higher, while the average length of stay was significantly shorter in city hospitals for these patients under services that may be undifferentiated, implying that consumption of resources at the county level was generally lower for treatment of similar conditions. This was consistent with the request of hospital function from the tiered healthcare delivery system policy that patients with a chronic disorder and acute patients in the rehabilitation phase were recommended to be under treatment in county-level hospitals, which normally demand longer inpatient time.

### 3.4. Quality Difference between the Two Levels in Each DRG

There were 444,951 observations left after excluding cases in high-middle and high-risk mortality groups, covering 205 DRGs. 34.8% of the cases were from city hospitals. Among the 592 dead cases, 164 were from city hospitals and mortality rate was 0.11%, 428 were from county hospitals with mortality rate of 0.15%. Differences in mortality and adverse events in each DRG were further analyzed between the two levels in these observations, and the results of significantly different DRGs are exhibited in Table 4.

Only in the group of infection or inflammation in the respiratory system without comorbidities (ES15) the mortality rate was significantly higher in county hospitals. There were 5493 cases in ES15. A total of 4369 were inpatient in county hospitals with 40 deaths, and 1124 in city hospitals without deaths observed. The most common principal diagnoses in this group were pulmonary infection, bronchopneumonia, and pneumonia. In the 40 death records in county hospitals, 29 patients were only coded as pulmonary infection without any information of location or type. In the remaining 11 patients, 2 were diagnosed with severe pneumonia, which had 1734 observations with 34 deaths in the whole sample. The city hospitals reported 30 deaths and county hospitals reported 4, whereas all of the other severe pneumonia dead patients had severe comorbidities except these two. Each case of dead patients in ES15 was diagnosed as atypical hyperplasia of the lung, persistent pneumonia, atypical pneumonia, and acute bronchitis, all of which had few cases with the same principal diagnosis in the whole sample, and among them, only these four patients died. There were three and two dead patients who were diagnosed with pneumonia and bronchopneumonia, respectively. Many patients had the same diagnoses in the whole sample and the mortality rate of them was 0.15% for pneumonia and 0.04% for bronchopneumonia, which were not very high; other patients died with the same diagnoses, except the two who had comorbidities or severe comorbidities.

Moreover, the infection or inflammation in respiratory system patients was divided into four DRGs in BJ-DRG based on the age of patients or their comorbidity status. No significant differences existed between city and county levels in the remaining three DRGs that had comorbidities, severe comorbidities, or were aged less than 17. According to the request of tiered healthcare delivery policy, pulmonary infection, bronchopneumonia, and pneumonia without comorbidities were common diseases that were recommended under therapy in county-level hospitals; however, it seems too early for county hospitals in the sample area taking care of patients with these conditions according to the significantly higher in-hospital mortality.

**Figure 2:**
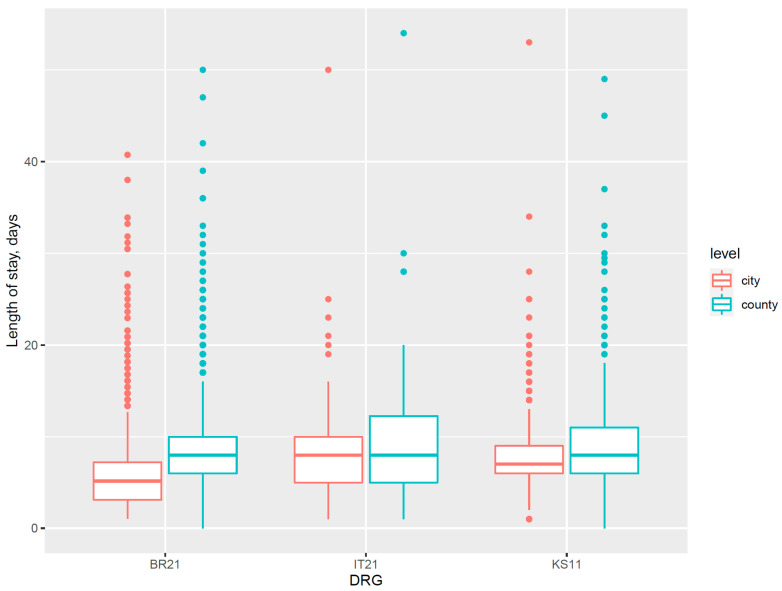
Box plot for differences in length of stay between city and county hospitals in DRG with a significant difference in pressure ulcer rate. DRG: diagnosis-related group; BR21: the DRG of cerebral ischemic diseases with severe comorbidities; IT21: the DRG of chronic inflammatory musculoskeletal and connective tissue disorders with severe comorbidities; KS11: the DRG of diabetes with severe comorbidities.

Differences in the quality of medical service were also revealed in the in-hospital adverse event category. There were 106 cases having pressure ulcers, 14 of which were from city hospitals and 92 from county hospitals. Patients in the DRG of cerebral ischemia with severe comorbidities (BR21), diabetes with severe comorbidities (KS11), and chronic inflammatory musculoskeletal and connective tissue disorders with severe comorbidities (IT21) had a significantly higher rate of pressure ulcers in county-level hospitals. These cases were all grouped into DRGs with severe comorbidities, and in the same DRG, the county hospitals also exhibited a generally longer length of stay than city level (Figure 2). This indicated the nursing competence for chronic diseases with severe comorbidities in county hospitals of sample area were probably required more improvement; thus, they frequently extended the length of stay of certain inpatients to achieve better outcomes.

There were 229 observations of adverse events caused by drugs in the remaining sample, with 72 in the city hospitals and 157 in the county. For patients with malignant proliferative diseases who underwent supportive treatment within 7 days (RU14) or committed follow-up examination after therapy (RW19), the rate of certain adverse events was significantly lower in city hospitals than in the county. The majority of cases in RW14 were in the principal diagnosis of chemotherapy for postoperative malignant tumor (at a proportion of 51.53% in city hospitals and 43.95% in the county) and chemotherapy course of tumor (18.35% in the city and 17.16% in the county). In RW19, the most common principal diagnosis was follow-up examination of malignant tumor after treatment (80.71% in the city and 79.60% in the county). The major adverse events displayed in the diagnosis fields of front sheets were myelosuppression after chemotherapy or medication. No significant differences were detected between the two levels in other DRGs.

Postoperative complications of the urogenital system were also significantly different, however, lower in county-level hospitals. Among the 28 cases with certain complications diagnosed, 27 were from city hospitals, of which 24 were in the DRG named surgery for carcinoma in situ and nonmalignant lesions (except ectopic pregnancy) other than uterine surgery with comorbidities (ND13), and diagnosis of their complications was diverticulum of uterine incision. This implied that surgery services for patients with carcinoma in situ such as endometrial polyps from city hospitals may have a higher risk of diverticulum of uterine incision, though the complication was simple and usually healed with half of a year.

No significant differences between city and county hospitals at the DRG level were found for other postoperative complications.

As a result, for patients who had conditions of respiratory infection or inflammation without comorbidities, cerebral ischemia with severe comorbidities, diabetes with severe comorbidities, chronic inflammatory musculoskeletal and connective tissue disorders with severe comorbidities, malignant proliferative diseases taking supportive treatment within 7 days, or malignant proliferative diseases committing follow-up examination after therapy, the quality of services in the county hospitals could be regarded as not high enough versus in city hospitals in the sample area.

### 3.5. Inpatient Cost Difference in City versus County Hospitals

The number of remained observations was 411,297 that covered 199 DRGs after excluding DRGs in which county hospitals had a significantly higher rate of mortality or in-hospital adverse events. The proportion of cases from city hospitals were 33.5% for these records. In these cases in which the quality of services from two levels seems indifferent, there were 129 DRGs whose total inpatient costs were significantly lower in county hospitals than in city hospitals. Among them, 57 DRGs were without comorbidities, 22 were DRGs that did not indicate whether comorbidities exist, 29 were with common comorbidities, 13 were with severe comorbidities, 7 were aged less than 17, and 1 malignant proliferative patient underwent supportive treatment within 30 days. The name for the DRGs with significantly lower cost in county hospitals and their average inpatient costs in each level of hospitals are demonstrated in the Supplementary Materials Table S1.

There were 261,596 cases in these 129 DRGs, which shared 39.5% of the total cases collected by the city platform. A total of 86,850 cases were from city hospitals admission, and 174,746 were from county hospitals. The average inpatient costs from these records were CNY 9450 (about USD 1443.96) at the city level, while CNY 5231 (USD 799.3) in the county, which was CNY 4229 (USD 646.2) lower than in city hospitals. Additionally, if patients in these DRGs were all admitted to county hospitals or their medical services were reimbursed at the same price (county hospital price level) for the same disease in different levels of hospitals, there would be CNY 265,043,865 (about USD 40.5 million) saved in medical costs, according to the 2019 data.

As a result, the diagnoses and operations in certain DRGs, especially those without comorbidities, could be the area where the further promotion of establishing tiered healthcare delivery system could be initiated, and the county hospitals could make more improvements in the quality of certain services to build up advantage or reduce their level of undifferentiation between hospital functions. On the other hand, medical insurance institutions could pay more attention to these DRGs in implementing the policy of the same price pay for the same disease in order to limit the irrational growth of healthcare expenditure.

## 4. Discussion

This study analyzed the medical record front sheets in one city of China in 2019 and discovered in 129 DRGs that the service quality of county-level hospitals versus city-level hospitals was not different, while the inpatient cost in county hospitals was significantly lower. Additionally, these cases were about 39.5% of the total observations, implying that there were cases with functional undifferentiation between the two levels of hospitals. As patients in China were not forced in their selection of admission in medical institutes, they might bypass the nearer and cheaper institutes and visit tertiary hospitals for the habit of thinking large hospital having better quality. Additionally, tertiary hospitals, which were city hospitals in this study, might receive patients with a common disease, ignoring their function and offering them overtreatment. Thus, it is necessary for further implementation of a tiered healthcare delivery policy to differentiate the function of hospitals.

Researchers have discussed the relationship between patients’ behavior and utilization of medical resources from many perspectives such as healthcare facility selection, payment methods, and patient flow. For example, Bell et al. discovered that women in Ghana were commonly bypassing nearest healthcare facilities while visiting farther hospitals for primary care services, for lacking confidence of clinician competence and less availability of supplies in primary care institutions [13]. They found that patients who went to the farther hospital for primary care paid extra cost (on average USD 18.5 vs. USD 10.1), which is similar to our results. Liang et al. analyzed 1 million observations in Taiwan for 17 years and discovered chronic patients with hypertension and diabetes would like most to bypass lower-level healthcare facilities and went straight to higher-level hospitals, which was consistent with our results [14]. Nguyen et al. assessed the quality of primary care at various types of health facilities from the perspective of patients’ satisfaction levels in Vietnam and found that community-based health centers provided better overall health outcomes [15]. Hamada et al. compared the quality, cost, and efficiency of services between patients with DRG-like prospective payment systems and fee-for-services by taking 8 year’s administrative data of AMI patients in Japan as an example, concluding that reform of prospective payment system would be of benefit in reducing healthcare resource consumption, but more discussion is still needed for the effect of service quality [16]. The main common point of this study and Hamada’s research was adopting DRG, whereas, unlike Hamada et al. study with a focus on the evaluation of payment system reform, this study mainly used DRG as a means of grouping patients by their clinical features to make them comparable between different levels of hospitals. Moreover, this study used similar indicators to Hamada et al. research on the evaluation of resource efficiency but used more indicators, including adverse events rate on the evaluation of the quality of services.

Although the Chinese government had proposed that establishing a tiered healthcare delivery system is a vital element of China’s healthcare reform [17], Chinese citizens were not forced to select certain medical institutions. Only policies of offering various cover ratios for patients admitting to different levels of hospitals (the highest cover ratio, which is the lowest self-payment ratio for patients in primary hospitals, and lowest cover ratio for patients in tertiary hospitals) were issued by some provincial medical insurance bureaus [18], of which the response was not satisfactory as the price in large-scale hospitals were originally higher [6,19].

As a result, it seemed more effective for Chinese health administrations to modify the behavior of medical providers than patients’ habits, whether through compulsory order or payment leverage. Additionally, to achieve that end, the public needed to know what kinds of medical services were inappropriate of being offered in tertiary hospitals. Zhao et al. [20] grouped medical record front sheets from 51 tertiary hospitals and 122 county hospitals in Shandong Province in 2018 and detected 26 DRGs from these cases, of which each institute could offer medical services. Afterward, they concluded that there would be about CNY 1.49 billion of health expenditure saved if 80% of patients from these 26 DRGs admitted in tertiary hospitals were referred downward into county hospitals. They conclude that tertiary hospitals offered too many services for patients with common disease which could get cued in lower healthcare institutes, which is same with our study, and the 26 DRGs in their results were also contained in the 129 DRGs. The main differences between the two studies were the principle of this study in detecting homogenized DRGs was more focused on comparing service quality between the two levels instead of service volume from each hospital, and the conclusion of this study was to offer a strategy of finding problem of undifferentiation of medical services and narrowing the range as much as possible, which indicated that the name of these 129 DRGs was not the most important and further policies of downward referral or “same price–same disease” should not cover all of these 129 DRGs. Although the study was a preliminary exploration of the function undifferentiation problem between general public hospitals in China based on one city medical record front sheet data, the method of selecting patients and narrowing diseases step by step had a value of promotion in ameliorating the situation of hospital function homogenization and decreasing the healthcare cost growth speed.

On the other hand, since the Leapfrog Group was founded in 2000, reporting hospital performance had been discussed as an instrument in improving consumers’ decision making [21]. Various metrics of comparing medical service quality and patient safety were developed and practiced including metrics based on voluntary self-report data such as Leapfrog Safe Practices Scores measures [22] and index with data compulsory reports such as hospital comparison website from Centers for Medicare and Medicaid Services [23]. Thus, disclosing information on DRGs that were not different in quality while cheaper in county hospitals than in city hospitals may encourage more patients with common diseases to choose lower-level institutes as their first choice of admission, which might also improve the decision-making status of certain patients.

### Limitations

There were several limitations in this study. First, the indicators of quality evaluation in this study were only focused on recent, in-hospital outcomes, for it was impossible with the sample data to find out whether a patient was readmitted once he or she was discharged. Thus, more detailed analysis with quality indicators of forwarding outcomes is essential for the local government or social institutes after a platform with the identity information of citizens is established. Second, there were still many problems in filling out the medical record front sheets. Many cases acquired from the City Healthcare Information Platform had problems such as missing a principal diagnosis or the code of diagnosis and operation not matching. Fortunately, no significant differences were detected for the distribution of these problemed cases between the two levels of hospitals; thus, no evidence of selection bias existed for the study conclusion (Supplementary Materials Table S2). Lastly, this study offered a strategy of finding patients who went to higher-level hospitals with unnecessary extra costs but not developed any model or methodology. There still needs further studies to form models with more detailed patients’ information and reliable indicators.

## 5. Conclusions

The study selected medical services that were cheaper in county hospitals while not different in their level of quality between city and county hospitals based on one city data from China and proposed a selection strategy with four steps. This strategy could help in locating patients who bypassed lower-level healthcare facilities and visited large hospitals unnecessarily, especially in countries where patients are free to access any level of medical institutes. Additionally, even in countries with a strict gatekeeping system, it could be applied to compare healthcare facilities across different levels of hospitals.

## Figures and Tables

**Table 1:** Baseline characteristics of sample hospitals.

Level	Hospital	Case NO.	DRGs	CMI	Average Cost, Thousand Yuan	Average Length of Stay, Days
City	1	114,591	723	1.04	12.38	6.8
	2	107,064	724	0.99	11.7	6.2
County	3	49,601	302	0.88	6.57	6.4
	4	23,150	284	0.61	11.42	8.7
	5	41,046	440	0.71	8.12	6.4
	6	57,203	635	0.81	7.42	7.2
	7	14,792	327	0.73	6.76	6.7
	8	19,952	162	0.64	5.81	6.9
	9	49,916	552	0.77	5.22	7.1
	10	37,453	304	0.68	5.94	6.6
	11	16,343	424	0.74	7.2	7.2
	12	32,815	280	0.62	5.85	6.7
	13	2757	151	0.72	6.46	7.0
	14	39,940	521	0.86	5.18	7.1

Case NO.: numbers of cases in each group; DRGs: diagnosis-related groups; CMI: case-mixed index.

**Table 2:** Other baseline characteristics of sample hospitals.

Level	Hospital	Cost Consumption Index	Time Consumption Index	Low-Risk MR (%)	Low-Middle Risk MR (%)	High-Middle Risk MR (%)	High-Risk MR (%)
City	1	0.69	0.83	0.02	0.26	1.67	7.58
	2	0.85	0.78	0.13	0.36	1.15	5.25
County	3	0.75	0.86	0.09	0.57	2.08	11.55
	4	1.27	1.08	0.06	0.27	2.34	14.44
	5	0.97	0.90	0.01	0.01	0.17	0
	6	0.63	0.90	0.05	0.27	2.19	10.30
	7	0.61	0.91	0.05	0.22	1.99	2.04
	8	0.80	0.96	0	0	0	0
	9	0.45	0.89	0.04	0.35	2.24	11.91
	10	0.67	0.87	0.19	0.63	3.41	5.85
	11	0.72	0.92	0.06	0.42	2.01	5.37
	12	0.72	0.97	0.05	0.67	1.11	40.09
	13	0.72	0.94	0	0.56	0.35	0
	14	0.43	0.83	0.05	0.28	1.47	11.09

MR: mortality rate.

**Table 3:** Differences between the two levels of hospitals in terms of mortality rate, cost, and length of stay.

Variables	Level	Case NO.	Distribution	*p* Value
Death	city	170,010	424 (0.25%)	<0.001
	county	322,496	1129 (0.35%)	
Cost, thousand yuan	city	170,010	6.39 (3.94, 9.52)	<0.001
	county	322,496	4.63 (2.74, 7.18)	
Length of stay, days	city	170,010	5 (3, 7)	<0.001
	county	322,496	6 (4, 9)	
Low-risk MR	city	62,235	46 (0.07%)	0.134
	county	136,624	75 (0.05%)	
Low-middle risk MR	city	38,398	118 (0.31%)	0.153
	county	98,272	353 (0.36%)	
High-middle risk MR	city	13,175	190 (1.44%)	0.020
	county	30,611	538 (1.76%)	
High-risk MR	city	1783	70 (3.93%)	<0.001
	county	1986	163 (8.21%)	

Categorical variables are shown as numbers (percentage), and continuous variables are shown as median (quantiles) in distribution. Case NO.: numbers of cases in each group; MR: mortality rate.

**Table 4:** The significant differences in the quality level between the two levels.

Indicator	DRG	RW	MR of DRG	Level	Number (Rate)	*p* Value	Average Length of Stay
Death	Infection or inflammation in respiratory system without comorbidities (ES15)	0.73	0.73%	city	0 (0%)	0.002	7.4
				county	40 (0.92%)		10.5
Pressure ulcers	Cerebral ischemic diseases with severe comorbidities (BR21)	1.28	0.47%	city	3 (0.15%)	0.005	8.1
				county	35 (0.77%)		9.1
	Chronic inflammatory musculoskeletal and connective tissue disorders with severe comorbidities (IT21)	0.94	0.27%	city	0 (0%)	0.001	7.8
				county	9 (6.29%)		11.1
	Diabetes with severe comorbidities (KS11)	0.89	0.21%	city	0 (0%)	0.034	7.6
				county	6 (0.33%)		11.0
Adverse event caused by drugs	Supportive treatment within 7 days for malignant proliferative patients (RU14)	0.37	0.01%	city	67 (0.59%)	<0.001	3.0
				county	137 (2.8%)		3.4
	Follow-up examination after therapy for malignant proliferative patients (RW19)	0.41	0.14%	city	5 (0.59%)	<0.001	4.0
				county	20 (3.68%)		7.3
Postoperative complications in urogenital system	Surgery for carcinoma in situ and nonmalignant lesions (except ectopic pregnancy) other than uterine surgery, with comorbidities (ND13)	0.6	0%	city	24 (7.55%)	0.007	5.8
				county	0 (0%)		7.7
	Other diseases of female reproductive system with comorbidities (NZ13)	0.28	0.11%	city	3 (2.16%)	0.010	3.6
				county	1 (0.13%)		9.0

The number of occurrences and their rates are shown in the number (rate) for each indicator. DRG: diagnosis-related group; RW: related weight; MR: mortality rate.

## Data Availability

The datasets generated and/or analyzed during the current study are available from the corresponding author on reasonable request.

## Supplementary Materials

The following are available online at https://www.mdpi.com/article/10.3390/healthcare9080922/s1, Table S1: DRGs with costs in county hospitals are significantly lower, Table S2: Reasons of exclusion between city- and county-level hospitals.

## Abbreviations

DRG: diagnosis-related group; BJ-DRG: Beijing version DRG splitter; ICD-10-CM: International Classification of Disease, Tenth Revision, Clinical Modification; ICD-9-CM: International Classification of Disease, Ninth Revision, Clinical Modification; RW: related weight; CMI: case mix index.
