# Supplementary material for: Detecting Undifferentiation of Tertiary and County Hospitals in China in Adoption of DRG Instrument
Source: Healthcare (Basel). 2021 Jul 21;9(8):922. doi: 10.3390/healthcare9080922 (PMC8393376; doi:10.3390/healthcare9080922)
Supplement: Supplementary file 1 [file healthcare-09-00922-s001.zip › healthcare-1201853-supplementary.pdf]

healthcare

MDPI

Table S1. DRGs with cost in county hospitals significantly lower.

DRG	RW	MR	Risk Group	Level	Inpatient Cost
Cerebral ischemic diseases with comorbidities (BR23)	0.97	0.12%	Low	city<br>county	9.02 ± 9.99<br>6.47 ± 6.04
Cerebral ischemic diseases without comorbidities (BR25)	0.74	0.19%	Low-middle	city<br>county	9.97 ± 12.28<br>6.43 ± 5.89
Other infections of the nervous system with severe comorbidities (BT21)	1.91	0.45%	Low-middle	city<br>county	12.3 ± 13.99<br>3.84 ± 7.59
Other infections of the nervous system without comorbidities (BT25)	1.8	0.13%	Low	city<br>county	9.04 ± 15.28<br>2.62 ± 5.52
Epilepsy with severe comorbidities (BV11)	1.55	0%	Zero-mortality	city<br>county	11.44 ± 11.11<br>6.88 ± 11.63
Epilepsy with comorbidities (BV13)	0.91	0.53%	Low-middle	city<br>county	9.48 ± 13.14<br>6.06 ± 7.36
Headache (BV39)	0.64	0%	Zero-mortality	city<br>county	5.44 ± 5.48<br>4.27 ± 1.85
Brain dysfunction without comorbidities (BX15)	1.54	0.22%	Low-middle	city<br>county	16.24 ± 22.85<br>8.59 ± 7.97
Peripheral neuropathy with comorbidities (BX23)	0.91	0.15%	Low	city<br>county	8.87 ± 11.87<br>5.35 ± 3.46
Peripheral neuropathy without comorbidities (BX25)	0.67	0.43%	Low-middle	city<br>county	7.85 ± 10.5<br>5.98 ± 6.81
Vitrectomy and retinal surgery (CB19)	0.55	0%	Zero-mortality	city<br>county	10.73 ± 6.24<br>6.8 ± 4.18
Lens surgery (CB39)	0.32	0%	Zero-mortality	city<br>county	6.88 ± 1.22<br>6.25 ± 3.16
Various types of cataract (CW19)	0.17	0%	Zero-mortality	city<br>county	7.95 ± 8.2<br>6.11 ± 2.39
Other diseases cause eye diseases (CX19)	0.77	0%	Zero-mortality	city<br>county	8.16 ± 8.03<br>5.36 ± 3.1
Other eye diseases without comorbidities (CZ15)	0.35	0%	Zero-mortality	city<br>county	8.96 ± 21.84<br>2.65 ± 2.48
Other external ear and ear surgery (DC29)	0.79	0%	Zero-mortality	city<br>county	6.3 ± 3.39<br>3.88 ± 1.62

Nasal cavity and sinus surgery (DD29)	0.81	0%	Zero-mortality	city<br>county	10.06 ± 5.18<br>8.41 ± 3.06
Tonsillectomy and / or adenoidectomy in age < 17 (DE20)	0.88	0%	Zero-mortality	city<br>county	7.75 ± 1.52<br>7.14 ± 1.26
Medium sized oral surgery without comorbidities (DG25)	0.64	0%	Zero-mortality	city<br>county	10.33 ± 7.12<br>5.77 ± 3.42
Imbalance and hearing impairment (DS19)	0.62	0.05%	Low	city<br>county	6.38 ± 9.4<br>4.2 ± 2.52
Otitis media and upper respiratory tract infection in age < 17 (DT10)	0.43	0.01%	Low	city<br>county	3.24 ± 3.4<br>1.68 ± 1.04
Otitis media and upper respiratory tract infection with comorbidities (DT13)	0.58	0%	Zero-mortality	city<br>county	11.19 ± 16.18<br>5.12 ± 6.34
Otitis media and upper respiratory tract infection without comorbidities (DT15)	0.31	0.14%	Low	city<br>county	10.94 ± 18.7<br>4.64 ± 3.88
Epiglottitis, laryngitis and tracheitis (DT29)	0.39	0%	Zero-mortality	city<br>county	7.09 ± 14.39<br>1.98 ± 1.41
Oral and dental diseases without comorbidities (DX15)	0.29	0.11%	Low	city<br>county	6.42 ± 10.91<br>3.33 ± 2.81
Major thoracic surgery without comorbidities (EB15)	2.29	0.30%	Low-middle	city<br>county	38.31 ± 21.94<br>32.99 ± 14.04
Respiratory infection / inflammation in age < 17 (ES10)	0.86	0.01%	Low	city<br>county	5.62 ± 4.77<br>2.48 ± 1.26
Chronic airway obstruction with severe comorbidities (ET11)	1.49	0.68%	Low-middle	city<br>county	13.47 ± 14.08<br>9.16 ± 10.36
Chronic airway obstruction without comorbidities (ET15)	0.8	0.40%	Low-middle	city<br>county	8.94 ± 9.73<br>7.24 ± 6.44
Respiratory symptoms and signs with comorbidities (EV13)	0.76	0.57%	Low-middle	city<br>county	10.05 ± 13.52<br>7.17 ± 9.61
Interstitial lung disease without comorbidities (EW25)	1.36	0.49%	Low-middle	city<br>county	8.5 ± 5.68<br>6.92 ± 7.02
Bronchitis and asthma in age < 17 (EX10)	0.48	0%	Zero-mortality	city<br>county	4.54 ± 3.84<br>2.24 ± 0.93
Bronchitis and asthma with comorbidities (EX13)	0.9	0.13%	Low	city<br>county	9.29 ± 15.91<br>6.14 ± 6.7
Bronchitis and asthma without comorbidities (EX15)	0.71	0.11%	Low	city<br>county	8.03 ± 9.33<br>5.22 ± 3.78

Atherosclerosis with comorbidities (FS23)	0.83	0.37%	Low-middle	city<br>county	9.66 ± 14.35<br>7.89 ± 10.96
Hypertension without comorbidities (FT25)	0.63	0.09%	Low	city<br>county	9.63 ± 14.46<br>6.84 ± 7.94
Valvular diseases without comorbidities (FU15)	0.85	0.77%	Low-middle	city<br>county	8.72 ± 11.22<br>5.91 ± 5.93
Arrhythmia and conduction disorder without comorbidities (FU35)	0.56	0.21%	Low-middle	city<br>county	7.72 ± 10.29<br>5.67 ± 6.38
Syncope and / or collapse without comorbidities (FV25)	0.65	0%	Zero-mortality	city<br>county	6.03 ± 5.87<br>4.54 ± 3.63
Other diseases of circulatory system with severe comorbidities (FZ11)	1.32	0.63%	Low-middle	city<br>county	11.68 ± 15.07<br>9.9 ± 12.79
Other diseases of circulatory system with comorbidities (FZ13)	1.3	0.60%	Low-middle	city<br>county	9.87 ± 11.31<br>6.52 ± 6.76
Other diseases of circulatory system without comorbidities (FZ15)	0.74	0.30%	Low-middle	city<br>county	6.33 ± 7.47<br>4.19 ± 5.13
Operation of anus and alimentary tract stoma with comorbidities (GC23)	0.84	0%	Zero-mortality	city<br>county	11.81 ± 7.08<br>7.28 ± 2.62
Operation of anus and alimentary tract stoma without comorbidities (GC25)	0.58	0%	Zero-mortality	city<br>county	9.08 ± 3.46<br>6.76 ± 2.04
Appendectomy with complex diagnosis without comorbidities (GD15)	0.63	0%	Zero-mortality	city<br>county	12.39 ± 2.48<br>10.26 ± 3.57
Inguinal and abdominal hernia surgery in age < 17 (GE10)	0.29	0%	Zero-mortality	city<br>county	6.99 ± 1.27<br>5.11 ± 1.42
Inguinal and abdominal hernia surgery with comorbidities (GE13)	0.5	0%	Zero-mortality	city<br>county	12.63 ± 5.74<br>9.8 ± 5.62
Inguinal and abdominal hernia surgery without comorbidities (GE15)	0.38	0.12%	Low	city<br>county	10.62 ± 11.32<br>8.39 ± 3
Inflammatory bowel disease (GT19)	0.81	0%	Zero-mortality	city<br>county	6.91 ± 6.2<br>4.31 ± 3.25
Esophagitis and gastroenteritis without comorbidities (GU15)	0.55	0.10%	Low	city<br>county	6.93 ± 8.48<br>4.92 ± 4.64
Peptic ulcer without comorbidities (GV29)	0.55	0.31%	Low-middle	city<br>county	7.93 ± 7.11<br>5.16 ± 4.24
Digestive tract obstruction or abdominal pain with comorbidities (GW13)	0.89	0.61%	Low-middle	city<br>county	9.9 ± 13.24<br>5.69 ± 7.6

Digestive tract obstruction or abdominal pain without comorbidities (GW15)	0.45	0.24%	Low-middle	city<br>county	8.52 ± 16.66<br>4.86 ± 5.55
Special diseases of digestive system (GX19)	0.91	0.22%	Low-middle	city<br>county	8.71 ± 9.02<br>3.4 ± 5.16
Other diseases of digestive system with comorbidities (GZ13)	0.64	0.19%	Low-middle	city<br>county	7.48 ± 10.44<br>5.26 ± 5.54
Other diseases of digestive system without comorbidities (GZ15)	0.38	0.11%	Low	city<br>county	8.72 ± 12.26<br>5.65 ± 4.2
acute pancreatitis with severe comorbidities (HT21)	1.12	0.77%	Low-middle	city<br>county	15.43 ± 15.06<br>9.59 ± 6.36
acute pancreatitis without comorbidities (HT25)	0.82	0.12%	Low	city<br>county	13.77 ± 12.21<br>7.55 ± 6.57
Acute biliary diseases with severe comorbidities (HU11)	1.9	0.47%	Low-middle	city<br>county	10.54 ± 12.34<br>8.6 ± 7.64
Acute biliary diseases with comorbidities (HU13)	0.74	0.14%	Low	city<br>county	10.76 ± 11.74<br>7.26 ± 6.54
Liver cirrhosis and alcoholic hepatitis without comorbidities (HV15)	0.74	0.73%	Low-middle	city<br>county	10.12 ± 10.33<br>7.34 ± 7.42
Other liver diseases with severe comorbidities (HZ11)	1.16	0%	Zero-mortality	city<br>county	9.38 ± 8.07<br>8.63 ± 11.82
Other liver diseases without comorbidities (HZ15)	0.72	0.14%	Low	city<br>county	8.69 ± 15.93<br>6.82 ± 7.19
Other diseases of biliary tract with comorbidities (HZ23)	0.67	0.42%	Low-middle	city<br>county	10.24 ± 9.62<br>8.72 ± 9.06
Major joint replacement without comorbidities (IC15)	2.1	0%	Zero-mortality	city<br>county	44.49 ± 11.21<br>33.04 ± 11.64
Upper limb long bone surgery in age < 17 (IF10)	0.62	0%	Zero-mortality	city<br>county	11.75 ± 6.27<br>10.66 ± 6.44
Upper limb long bone surgery with comorbidities (IF13)	1.38	0%	Zero-mortality	city<br>county	24.75 ± 17<br>18.39 ± 6.38
Upper limb long bone surgery without comorbidities (IF15)	0.96	0%	Zero-mortality	city<br>county	16.54 ± 11.49<br>13.23 ± 6.32
Lower limbs long bone surgery with comorbidities (IF23)	2.5	0%	Zero-mortality	city<br>county	37.39 ± 18.99<br>25.98 ± 11.52
Lower limbs long bone surgery without comorbidities (IF25)	1.4	0.22%	Low-middle	city<br>county	27.93 ± 20.85<br>20.17 ± 10.52

Muscle and tendon surgery without comorbidities (IG15)	0.47	0%	Zero-mortality	city<br>county	10.05 ± 11.83<br>6.22 ± 7.09
Transplantation or debridement without comorbidities (IH25)	0.56	0%	Zero-mortality	city<br>county	8.83 ± 8.99<br>7.52 ± 7.42
Removal of fixator for long bone and facet joint without comorbidities (IH35)	0.43	0%	Zero-mortality	city<br>county	6.45 ± 3.26<br>5.95 ± 2.97
Chronic inflammatory musculoskeletal and connective tissue disorders with comorbidities (IT23)	0.87	0%	Zero-mortality	city<br>county	8.69 ± 11.74<br>6.53 ± 6.45
Chronic inflammatory musculoskeletal and connective tissue disorders without comorbidities (IT25)	0.58	0.09%	Low	city<br>county	8.71 ± 14.45<br>7.08 ± 11.7
Total mastectomy for malignant breast tumor without comorbidities (JA15)	1.1	0%	Zero-mortality	city<br>county	18.79 ± 7.56<br>15.53 ± 2.61
Ulceration and cellulitis with comorbidities (JD25)	0.83	0%	Zero-mortality	city<br>county	10.94 ± 11.71<br>6.49 ± 4.25
Other operations of skin and subcutaneous tissue with comorbidities (JJ13)	0.65	0%	Zero-mortality	city<br>county	12.44 ± 13.91<br>5.09 ± 3.53
Other operations of skin and subcutaneous tissue without comorbidities (JJ15)	0.37	0.05%	Low	city<br>county	7.79 ± 8.04<br>4.48 ± 2.82
Malignant tumor of breast without comorbidities (JR15)	0.81	0.45%	Low-middle	city<br>county	9.53 ± 10.87<br>5.02 ± 5.82
Severe skin disease with comorbidities (JV13)	1.1	0.44%	Low-middle	city<br>county	10.11 ± 18.57<br>5.08 ± 5.87
Severe skin disease without comorbidities (JV15)	0.65	0.36%	Low-middle	city<br>county	8.88 ± 14.56<br>4.39 ± 7.11
Dermatitis or eczema (JV29)	0.56	0.37%	Low-middle	city<br>county	7.8 ± 13.55<br>2.83 ± 2.42
Cellulitis and other infectious skin diseases without comorbidities (JV35)	0.68	0.11%	Low	city<br>county	8.45 ± 11.8<br>3.94 ± 4.03
Mild skin diseases without comorbidities (JV45)	0.41	0%	Zero-mortality	city<br>county	7.01 ± 16.42<br>3.55 ± 3.65
Major thyroidectomy (KD19)	0.86	0.23%	Low-middle	city<br>county	17.79 ± 6.69<br>13.25 ± 3.25
Endocrine disorders with severe comorbidities (KT11)	0.82	0.27%	Low-middle	city<br>county	9.77 ± 19.58<br>5.8 ± 4.9
Other urological operations without comorbidities (LJ15)	0.48	0%	Zero-mortality	city<br>county	11.11 ± 8.25<br>6.87 ± 6.98

Nephritis and nephropathy with severe comorbidities (LS11)	0.94	0.31%	Low-middle	city<br>county	9.36 ± 11.45<br>5.84 ± 3.74
Nephritis and nephropathy with comorbidities (LS13)	0.8	0.17%	Low-middle	city<br>county	8.03 ± 10.88<br>3.56 ± 3.06
Urinary tract infection with comorbidities (LU13)	0.87	0.19%	Low-middle	city<br>county	8.87 ± 11.51<br>5.34 ± 9
Urinary tract infection without comorbidities (LU15)	0.56	0%	Zero-mortality	city<br>county	6.09 ± 6.23<br>4.44 ± 4.91
Urinary calculi or obstruction and urethral stricture (LW19)	0.38	0.03%	Low	city<br>county	6.53 ± 11.77<br>3.29 ± 4.01
Other diseases of urinary system without comorbidities (LZ15)	0.34	0%	Zero-mortality	city<br>county	6.89 ± 7.17<br>5.28 ± 4.96
Surgery for non-malignant lesions of testis in age < 17 (MB10)	0.35	0%	Zero-mortality	city<br>county	7.08 ± 1.43<br>5.42 ± 1.43
Prostatectomy with comorbidities (MC13)	1.34	0%	Zero-mortality	city<br>county	20.43 ± 4.46<br>17.99 ± 4.22
Other male reproductive system surgery without comorbidities (MJ15)	0.31	0%	Zero-mortality	city<br>county	6.58 ± 4.14<br>5.01 ± 2.2
Other diseases of male reproductive system (MZ19)	0.63	0.19%	Low-middle	city<br>county	7.42 ± 13.55<br>5.68 ± 6.22
Reconstruction of female reproductive system (NB19)	0.86	0%	Zero-mortality	city<br>county	18.17 ± 6.59<br>11.41 ± 3.28
Uterine surgery for carcinoma in situ and non-malignant lesions (except ectopic pregnancy) without comorbidities (NC15)	0.81	0%	Zero-mortality	city<br>county	14.33 ± 3.11<br>12.53 ± 3.85
Surgery for carcinoma in situ and non-malignant lesions (except ectopic pregnancy) other than uterine surgery with comorbidities (ND13)	0.6	0%	Zero-mortality	city<br>county	11.06 ± 6.69<br>9.43 ± 3.13
Operation of vulva, vagina and cervix (NE19)	0.31	0%	Zero-mortality	city<br>county	7.76 ± 4.98<br>4.95 ± 2.97
Malignant tumor of female reproductive system without comorbidities (NR15)	0.81	0.57%	Low-middle	city<br>county	15.92 ± 25.83<br>7.36 ± 6.32
Cesarean section with uterine and / or adnexal surgery (OB19)	0.84	0.05%	Low	city<br>county	9.24 ± 4.81<br>9.08 ± 4.36
Cesarean section with severe comorbidities (OB21)	0.99	0.13%	Low	city<br>county	11.49 ± 7.32<br>10.5 ± 8.83
Vaginal delivery with surgery with comorbidities (OC13)	0.6	0%	Zero-mortality	city<br>county	5.66 ± 3.95<br>4.59 ± 1.75

Vaginal delivery with surgery without comorbidities (OC15)	0.54	0.08%	Low	city	5.39 ± 4.27
				county	3.94 ± 1.39
Abortion with cervical dilatation and curettage or hysterectomy without comorbidities (OD35)	0.17	0%	Zero-mortality	city	5.96 ± 3.79
				county	1.91 ± 1.1
Abortion outside operating room without comorbidities (OS25)	0.24	0.09%	Low	city	4.32 ± 5.47
				county	1.86 ± 1.13
Preterm or false labor with comorbidities (OU13)	0.45	0%	Zero-mortality	city	3.09 ± 3.95
				county	1.28 ± 0.87
Preterm or false labor without comorbidities (OU15)	0.31	0%	Zero-mortality	city	1.81 ± 1.3
				county	1.34 ± 0.86
Term birth (weight > 2499g) with serious problems (PU11)	0.88	0.05%	Low	city	4.27 ± 4.6
				county	2.31 ± 1.18
Term birth (weight > 2499g) with other problems (PU13)	0.69	0%	Zero-mortality	city	4.13 ± 2.19
				county	2.96 ± 1.87
Abnormal hemogram with comorbidities (QS13)	0.49	0.50%	Low-middle	city	8.78 ± 11.97
				county	5.78 ± 4.89
Coagulation disorders (QT19)	0.8	0%	Zero-mortality	city	6.37 ± 8.14
				county	3.98 ± 4.19
Supportive treatment of malignant proliferative diseases within 3 days (RU12)	1.35	0.03%	Low	city	15.04 ± 14.67
				county	8.95 ± 5.14
Radiotherapy and other treatments for malignant proliferative diseases (RV19)	2.6	0%	Zero-mortality	city	30.35 ± 24.64
				county	19.03 ± 9.93
Sepsis with severe comorbidities (SR11)	3.19	0.19%	Low-middle	city	8.99 ± 14.3
				county	5.7 ± 11.04
Sepsis without comorbidities (SR15)	2.1	0.17%	Low-middle	city	4.24 ± 9.03
				county	2.05 ± 2.22
Viral diseases with comorbidities (ST13)	1.28	0%	Zero-mortality	city	8.51 ± 10.82
				county	2.74 ± 2.23
Other infectious and parasitic diseases with severe comorbidities (SZ11)	2.14	0.17%	Low-middle	city	11.49 ± 16.03
				county	3.86 ± 5.63
Other infectious and parasitic diseases with comorbidities (SZ13)	1.29	0%	Zero-mortality	city	8.51 ± 6.24
				county	3.37 ± 2.76
Other infectious and parasitic diseases without comorbidities (SZ15)	0.43	0%	Zero-mortality	city	7.91 ± 5.44
				county	3.73 ± 2.91
Injury related debridement without comorbidities (VC15)	0.52	0%	Zero-mortality	city	18.46 ± 18.1
				county	6.85 ± 9.34

Hand injury surgery (VD19)	0.77	0%	Zero-mortality	city<br>county	22.62 ± 29.58<br>6.41 ± 3.16
Damage without comorbidities (VR15)	0.54	0.23%	Low-middle	city<br>county	7.05 ± 7.9<br>5.52 ± 6.72
Toxic reaction of drugs and other substances without comorbidities (VS25)	0.53	0.74%	Low-middle	city<br>county	5.37 ± 6.06<br>5 ± 9.02
Other burn without comorbidities (WZ15)	0.5	0.28%	Low-middle	city<br>county	5.21 ± 2.85<br>4.28 ± 9.13
Rehabilitation without comorbidities (XR15)	1.27	0%	Zero-mortality	city<br>county	24.93 ± 22.63<br>10.34 ± 11.38

DRGs only with cost in county hospitals significantly lower were displayed at this table. DRG: diagnosis related group; RW: related weight; MR: mortality rate.

Table S2. Reasons of exclusion between two level hospitals.

Reason	Level	Total Number of Cases	Distribution	p Value
Newborn cases without weight information	city<br>county	10,189<br>45,384	295 (2.9%)<br>1309 (2.88%)	0.978
Missing or irregulated coding of principle diagnosis	city<br>county	10,189<br>45,384	6373 (62.55%)<br>28,037 (61.78%)	0.151
Total in-hospital cost lower than 50 yuan	city<br>county	10,189<br>45,384	1881 (18.46%)<br>8361 (18.42%)	0.939
Length of stay over 60 days	city<br>county	10,189<br>45,384	1109 (10.88%)<br>5048 (11.12%)	0.499
Codes of diagnosis and operation not match	city<br>county	10,189<br>45,384	357 (3.5%)<br>1625 (3.58%)	0.728
Diagnosis of pregnancy with age over 50	city<br>county	10,189<br>45,384	276 (2.71%)<br>1263 (2.78%)	0.705
Palliative treatment for malignant tumor cases	city<br>county	10,189<br>45,384	172 (1.69%)<br>673 (1.48%)	0.138

Number of cases occurred and percentage in each level of hospitals for the reasons were displayed as Distribution.
